# Genomic prediction of host resistance to sea lice in farmed Atlantic salmon populations

**DOI:** 10.1186/s12711-016-0226-9

**Published:** 2016-06-29

**Authors:** Hsin-Yuan Tsai, Alastair Hamilton, Alan E. Tinch, Derrick R. Guy, James E. Bron, John B. Taggart, Karim Gharbi, Michael Stear, Oswald Matika, Ricardo Pong-Wong, Steve C. Bishop, Ross D. Houston

**Affiliations:** The Roslin Institute and Royal (Dick) School of Veterinary Studies, The University of Edinburgh, Midlothian, EH25 9RG UK; Landcatch Natural Selection Ltd., 15 Beta Centre, Stirling University Innovation Park, Stirling, FK9 4NF UK; Institute of Aquaculture, University of Stirling, Stirling, FK9 4LA UK; Edinburgh Genomics, Ashworth Laboratories, King’s Buildings, University of Edinburgh, Edinburgh, EH9 3JT UK; Institute of Biodiversity, Animal Health and Comparative Medicine, University of Glasgow, Bearsden Road, Glasgow, G61 1QH UK

## Abstract

**Background:**

Sea lice have significant negative economic and welfare impacts on marine Atlantic salmon farming. Since host resistance to sea lice has a substantial genetic component, selective breeding can contribute to control of lice. Genomic selection uses genome-wide marker information to predict breeding values, and can achieve markedly higher accuracy than pedigree-based methods. Our aim was to assess the genetic architecture of host resistance to sea lice, and test the utility of genomic prediction of breeding values. Individual lice counts were measured in challenge experiments using two large Atlantic salmon post-smolt populations from a commercial breeding programme, which had genotypes for ~33 K single nucleotide polymorphisms (SNPs). The specific objectives were to: (i) estimate the heritability of host resistance; (ii) assess its genetic architecture by performing a genome-wide association study (GWAS); (iii) assess the accuracy of predicted breeding values using varying SNP densities (0.5 to 33 K) and compare it to that of pedigree-based prediction; and (iv) evaluate the accuracy of prediction in closely and distantly related animals.

**Results:**

Heritability of host resistance was significant (0.22 to 0.33) in both populations using either pedigree or genomic relationship matrices. The GWAS suggested that lice resistance is a polygenic trait, and no genome-wide significant quantitative trait loci were identified. Based on cross-validation analysis, genomic predictions were more accurate than pedigree-based predictions for both populations. Although prediction accuracies were highest when closely-related animals were used in the training and validation sets, the benefit of having genomic-versus pedigree-based predictions within a population increased as the relationships between training and validation sets decreased. Prediction accuracy reached an asymptote with a SNP density of ~5 K within populations, although higher SNP density was advantageous for cross-population prediction.

**Conclusions:**

Host resistance to sea lice in farmed Atlantic salmon has a significant genetic component. Phenotypes relating to host resistance can be predicted with moderate to high accuracy within populations, with a major advantage of genomic over pedigree-based methods, even at relatively sparse SNP densities. Prediction accuracies across populations were low, but improved with higher marker densities. Genomic selection can contribute to lice control in salmon farming.

**Electronic supplementary material:**

The online version of this article (doi:10.1186/s12711-016-0226-9) contains supplementary material, which is available to authorized users.

## Background

Genomic selection (GS) involves the prediction of individual breeding values for complex traits by combining statistical methods with genome-wide single nucleotide polymorphism (SNP) data. Relationships between SNPs and traits of interest are first determined within a reference (or training) population, and then they are used to identify selection candidates with high genetic merit in the absence of phenotype records [1, 2]. The feasibility of GS schemes depends on the availability of a high-quality SNP genotyping platform and on extensive trait records collected in the reference populations. Due to the increased availability of high-density SNP chips and the development of genotyping-by-sequencing for several economically important livestock and aquaculture species (e.g. [3–7]), GS has become a widely used approach, particularly for traits of economic and welfare importance (e.g. disease resistance). The accuracy of predicted breeding values based on genomic data is expected to be substantially higher than that based on pedigree records alone, but depends on many variables, including the genetic architecture of the trait, SNP density, sample size, and the degree of relationship between the reference and validation sets [8, 9].

In Atlantic salmon farming, ectoparasitic copepods, commonly known as sea lice (specifically *Lepeophtheirus salmonis* in Europe and *Caligus rogercresseyi* in Chile), are the primary threat to sustainable production, and have a negative economic, animal welfare, and environmental impact. Symptoms of *L. salmonis* infection include skin lesions, osmotic imbalance, and increased susceptibility to other infections as a result of host immune suppression and skin damage [10]. Frequent chemical treatments are required to control louse infections on commercial farms and result in large annual costs, potential environmental damage, and a high prevalence of drug-resistant lice [10, 11]. However, there is encouraging evidence from challenge trials that revealed heritabilities of approximately 0.2 to 0.3 for lice resistance, as measured by counts of lice on the fish (e.g. [11–14]), which highlights host genetic variation in resistance to lice. Therefore, selective breeding to improve host resistance to lice in farmed salmon populations is an increasingly important component of disease control [9, 11]. Given the importance of the sea lice issue to the salmon industry, this trait is also a high priority candidate for GS to accelerate the production of stocks with increased resistance.

The quantitative genetic models that underpin GS can be broadly split into two categories based on the assumptions that underlie the genetic architecture of the trait. The first category assumes an even distribution of the genetic variance across the genome and includes genomic best linear unbiased prediction (GBLUP) methods. The second category allows for heterogeneity in the contribution of the markers to the genetic variance, which is typically modelled using Bayesian methods (e.g. [15]). While the Bayesian methods (e.g. Bayes B) are generally more accurate than GBLUP on simulated data, particularly when the number of quantitative trait loci (QTL) that underlie the genetic variance is small [8], prediction accuracy using ‘experimental’ data in livestock breeding schemes is often very similar with either of these two methods [16]. Genomic prediction using these models relies both on capturing linkage disequilibrium (LD) between SNPs and QTL and on accurate estimates of realised genetic relationships between individuals [9, 17]. In typical farm animal populations, prediction accuracy depends largely on the latter [18], but the persistency of prediction accuracy across generations and between unrelated populations depends on the LD between SNPs and QTL [2, 9, 17]. For most commercial aquaculture breeding programmes, the availability of large full-sib families facilitates extensive trait measurements on individuals that are closely related to the selection candidates. Therefore, within-population genomic prediction will capitalise on realised genetic relationships, and the role of LD between SNPs and QTL may be less crucial [9, 18]. However, for salmon with a discrete 3-or 4-year generation interval, accuracy of prediction across adjacent year groups with limited genetic connectivity between them will depend more on LD, and is likely more challenging.

Family-based selective breeding programmes for Atlantic salmon have traditionally focused on economically important traits that can be easily measured on the selection candidates (e.g. growth) and on traits that can be measured on close relatives (e.g. full and half siblings), such as disease resistance and processing traits. Studies of GS in aquaculture using both simulated and ‘experimental’ data have suggested that genomic prediction can result in more accurate breeding values than traditional pedigree-based approaches (e.g. [9, 19–21]). However, the cost-efficiency of GS is critical; both high-density SNP arrays and extensive collection of trait data can be prohibitively expensive for routine genomic evaluations. Therefore, knowledge of the optimal design of reference populations and of the required SNP density is important, as well as quantification of the benefit that can be expected from the implementation of GS.

The objectives of this study were to (i) estimate the heritability of host resistance to sea lice using both genomic and pedigree-based methods, (ii) analyse the genetic architecture of host resistance by performing a GWAS, (iii) assess the accuracy of genomic prediction using various SNP densities up to 33 K SNPs and compare it to that of pedigree-based prediction, and (iv) test genomic prediction accuracies in closely and more distantly related reference and validation populations.

## Methods

### Animals and challenge experiment

The animals used in the study originated from a commercial Atlantic salmon breeding programme (Landcatch, UK). Due to the 4-year generation interval, the breeding program consists of four sub-populations (referred to as year groups), two of which were studied. Full details for population I (2007 year group, n = 624) were previously described in Tsai et al. [21]. Briefly, this population consisted of 531 genotyped offspring with complete phenotype and genotype information, derived from 61 nucleus families (30 sires and 59 dams). The families in population I were reared in separate tanks until approximately 9 months post-hatch, at which time they were mixed. Population II (2010 year group, n = 874) comprised 151 families (98 sires and 188 dams), with 588 offspring that were phenotyped and genotyped. The families in population II were mixed at first feeding and reared in a single common tank. The lice challenge trials were conducted at the Marine Environmental Research Laboratory (Machrihanish, UK) in 2007 and 2010, respectively. The challenge protocols were similar for both populations; the fish (approximately 1 year post-hatching) were challenged in a single tank with a moderate dose of copepodid larvae (90 to 96 larvae per fish) and then monitored daily until most lice had moulted into chalimus I. Sampling and measurements began on day 7 post-challenge and lasted 1 and 4.5 days for populations I and II, respectively (for population I, lice counts were shown to be stable between 7 and 17 days post-challenge [11]). Prior to lice counting, fish were euthanized with benzocaine as described in Gharbi et al. [11]. Phenotypes including weight (g), length (mm), and sea lice count [number of sea lice per fish, measured using a stereo-microscope (Olympus SZ-40)] were recorded for each fish. An adipose fin clip was collected and stored in ethanol for DNA extraction. For population I, pedigree information for each individual was traced by using passive integrated transponder (PIT) tags. For population II, a standard parentage assignment panel of 108 SNPs was screened on a Sequenom platform (DNA LandMarks Inc., Canada) to construct the pedigree.

All animals were reared in accordance with relevant national and EU legislation concerning health and welfare. The challenge experiment was performed by the Marine Environmental Research Laboratory (Machrihanish, UK) under approval of the ethics review committee of the University of Stirling (Stirling, UK) and according to Home Office license requirements. Landcatch are accredited participants in the RSPCA Freedom Foods standard, the Scottish Salmon Producers Organization Code of Good Practice, and the EU Code-EFABAR Code of Good Practice for Farm Animal Breeding and Reproduction Organizations.

### SNP genotyping

DNA was extracted from fin tissue samples using the DNeasy 96 tissue DNA extraction kit (Qiagen, UK). Population I was genotyped with an Affymetrix Axiom SNP array that included ~132 K SNPs [22] and population II was genotyped with the custom Affymetrix Axiom ~35 K array described in Tsai et al. [21]. This 35 K array is used for routine genomic evaluations and includes a subset of high-quality SNPs of the 132 K array that were selected based on having a good distribution throughout the genome and minimal LD between pairs of SNPs [21]. Sex of the fish was predicted by using the Y-specific probes on the 132 K array, as described by Houston et al. [22]. Filtering of SNP data was performed using the Plink software [23], excluding SNPs with Mendelian errors, SNPs with a minor allele frequency (MAF) lower than 0.1 and SNPs with a proportion of missing genotypes greater than 0.03. Finally, approximately 33 K SNPs were retained for analyses in both populations.

### Genetic parameters for lice resistance

#### Data normalization

The raw data for lice counts showed a positively skewed distribution (See Additional file 1: Figure S1), thus to normalize this distribution, we transformed the data using a previously applied approach that also accounts for an approximation of the surface area of the fish [13]:

1 $$\log_{\text{e}} {\text{LD}} = \log_{\text{e}} \left( {\left( {{\text{LC}} + 1} \right)/\left( {\text{BW}} \right)^{2/3} } \right),$$

where ${\text{LC}}$ is the number of lice counted on the fish (plus 1 to avoid a computation error since some fish may have zero lice), $\left( {\text{BW}} \right)^{2/3}$ is an approximation of the whole surface of the skin of each individual, where ${\text{BW}}$ represents the body weight (g) at the time of the sea lice challenges. A moderate correlation of 0.35 was found between body surface and lice count.

#### Estimation of genetic parameters

The heritability of host resistance to sea lice count (and of weight and length traits) was estimated using both genomic and pedigree-based analyses for the two populations. Only fish with complete phenotype and genotype records were included, resulting in 531 and 588 fish in populations I and II, respectively. Heritabilities were estimated by ASReml 3.0 [24] using genomic and pedigree-based relationship matrices (**G**-matrix and **A**-matrix, respectively) with the following mixed model:

2 $${\mathbf{y}} =\upmu + {\mathbf{Xb}} + {\mathbf{Za}} + {\mathbf{e}},$$

where ${\mathbf{y}}$ is a vector of observed phenotypes, $\upmu$ is the overall mean of phenotype records, ${\mathbf{b}}$ is the vector of fixed effects, ${\mathbf{a}}$ is a vector of additive genetic effects distributed as ~${\text{N}}\left( {0,{\mathbf{G}}\upsigma_{\text{a} }^{2} } \right)$ or ${\text{N}}\left( {0,{\mathbf{A}}\upsigma_{\text{a}}^{2} } \right)$ where $\upsigma_{\text{a}}^{2}$ is the additive (genetic) variance, ${\mathbf{G}}$ and ${\mathbf{A}}$ are the genomic and pedigree relationship matrices, respectively. ${\mathbf{X}}$ and ${\mathbf{Z}}$ are the corresponding incidence matrices for fixed and additive effects, respectively, and **e** is a vector of residuals. If the SNPs applying sex as the fixed effect did not surpass the genome-wide significance threshold (Bonferroni correction (0.05/N), where N represents the number of QC-filtered SNPs across the entire genome), it was omitted from subsequent analyses. The genomic relationship matrix was constructed by the Genabel R package [25] using the method of VanRaden [26] and then inverted by applying a standard R function (https://www.r-project.org/). Narrow sense heritability was estimated as the ratio of additive genetic variance to total phenotypic variance. Phenotypic correlations between traits were estimated using ASReml 3.0 [24] and genetic correlations were estimated using bivariate analyses implemented in ASReml 3.0 [24] as well.

### Genome-wide association study

The two-step ‘GRAMMAR’ approach was used to perform the GWAS using the GenABEL R Package [25]. The GWAS was performed in each population separately, and on the two populations combined. First, model (2) was applied to adjust the lice count data based on fixed (year group in the combined analysis) and polygenic effects (relationships between animals as measured by the genomic relationship matrix). Subsequently, the mmscore method [27] of GenABEL was applied to measure the association between individual SNPs and the residuals from model (2) (which are corrected for family relatedness). Significance thresholds were calculated using a Bonferroni correction to obtain genome-wide (0.05/number of all quality-control filtered SNPs, ~33 K) and chromosome-wide (0.05/number of SNPs on the corresponding chromosome) thresholds, respectively. For the SNPs that were closest to chromosome-wide significance (i.e. those with the lowest P values), allele substitution effects were estimated using model (2) in ASReml 3.0 [24] by including the fixed effects of SNP genotype and population. The additive effect (*a*) of the SNP was calculated as half the difference between the predicted phenotypic means of the two homozygotes, i.e. $\left( {{\text{AA}} - {\text{BB}}} \right)/2$, and the dominance effect (*d*) was calculated as ${\text{AB}} - \left[ {\left( {{\text{AA}} + {\text{BB}}} \right)/2} \right]$, where the ${\text{AB}}$ represents the predicted phenotypic mean of the heterozygote. The additive genetic SNP variance $(\sigma_{SNP}^{2})$ was estimated using the following equation:

3 $$\sigma_{SNP}^{2} = 2{\text{pq}}\left( {a + d\left( {{\text{q}} - {\text{p}}} \right)} \right)^{2} ,$$

where p and q are the frequency of the major and minor alleles at the SNP, respectively. The proportion of variance explained by the SNP is then given by:

4 $$\sigma_{SNP}^{2} /\sigma_{a}^{2} ,$$

where $\sigma_{a}^{2}$ is the total additive genetic variance of the trait when no SNP effects are included in the model.

### Assessment of genomic prediction

The utility of genomic prediction for resistance to lice was assessed by cross-validation analyses under various scenarios (see below) in which (i) varying SNP densities (0.5, 1, 5, 10, 20 K (all chosen at random), and 33 K (full dataset)) and (ii) varying degrees of relationships between training and validation sets were applied.

#### Scenario (i): Random selection

Within each population (which correspond to discrete ‘year groups’ of a commercial Atlantic salmon breeding programme), cross-validation analysis was performed by selecting five random non-overlapping training and validation sets as described previously [21]. At each SNP density (0.5 to 33 K SNPs), GBLUP was applied to predict the masked phenotypes of the validation sets and the resulting prediction accuracy was compared to that of pedigree-based BLUP (PBLUP), as described above. The average accuracy across the five cross-validation replicates for each SNP density was computed.

#### Scenario (ii): Sibling

Within each population, training and validation sets were established such that both sets contained representatives of each family. The same cross-validation analyses were performed as for Scenario (i).

#### Scenario (iii): Non-sibling

Within each population, training and validation sets were established such that full siblings were not included in either set (i.e. different full-sibling families were used for training and validation sets). The resulting training and validation sets were more distantly related than for Scenarios (i) and (ii), although they did contain some half-sibs. The same cross-validation analyses were performed as for Scenarios (i) and (ii).

#### Scenario (iv): Across populations

To assess prediction accuracy across populations per year group, population I was used as the training set and population II as the validation set, and vice versa. The same genomic prediction and cross-validation analyses were performed as for Scenarios (i) to (iii), but pedigree-based prediction was not possible since genetic links between the two populations were absent from the available pedigree.

#### Cross-validation

The five-fold cross-validation analyses for each scenario described above were performed using the methods described in Tsai et al. [21]. Briefly, for the within-population analyses, populations I and II were each divided into a training (80 %) and validation (20 %) set. Phenotypes (i.e. lice counts) of the samples in the validation sets were then masked and GBLUP or pedigree-based BLUP (PBLUP) was applied to predict the phenotypes of the masked individuals using model (2) implemented in ASReml 3.0 [24]. The Pearson correlation coefficient of the estimated breeding values (EBV) [either genomic EBV (GEBV) or pedigree-based EBV (PEBV)] with the adjusted phenotype of the masked validation set. Accuracy was calculated as the correlation divided by the square root of the heritability using all individuals, and then averaged across the five replicates (Figs. 2, 3).

## Results

### General statistics and genetic parameters of resistance to lice and growth

Estimated heritability for lice count was moderate (~0.3) and relatively consistent when using a pedigree relationship matrix (Table 1). Estimates of heritability for the growth-related traits (weight and length) were higher (~0.6), in line with previous estimates [21]. The two growth traits had high positive phenotypic and genetic correlations with each other (~0.93 to 0.96), and correlations of the growth traits with lice count were either equal to zero or slightly negative (Table 2).

**Table 1:** General statistics and heritability estimates for lice count and growth traits

	Population I		Population II	
	Mean (SD)	Heritability^a^ (SE)	Mean (SD)	Heritability^a^ (SE)
Lice^b^	25.8 (12.3)	0.33 (0.08)/0.27 (0.08)	18.3 (9.1)	0.22 (0.06)/0.27 (0.08)
Length	214.2 (16.1)^c^	0.61 (0.07)/0.51 (0.11)^c^	206.2 (14.3)	0.51 (0.07)/0.50 (0.10)
Weight	112.0 (21.0)^c^	0.61 (0.07)/0.49 (0.10)^c^	89.9 (19.9)	0.50 (0.07)/0.50 (0.10)

SD is the standard deviation and SE is the standard error

^a^Heritability was estimated based on the G-matrix/A-matrix

^b^The lice count data (number of lice per fish) used here was without data adjustment

^c^The results are from Tsai et al. [21]

**Table 2:** Estimates of genetic and phenotypic correlations between lice count and growth traits in populations I and II

Genetic correlation	Phenotypic correlation		
	Lice	Length	Weight
*Population I*			
Lice	–	−0.04	−0.06
Length	0.10	–	0.96
Weight	0.11	0.96	–
*Population II*			
Lice	–	−0.1	−0.1
Length	−0.3	–	0.93
Weight	−0.3	0.95	–

### Genome-wide association study

**Fig. 1:**
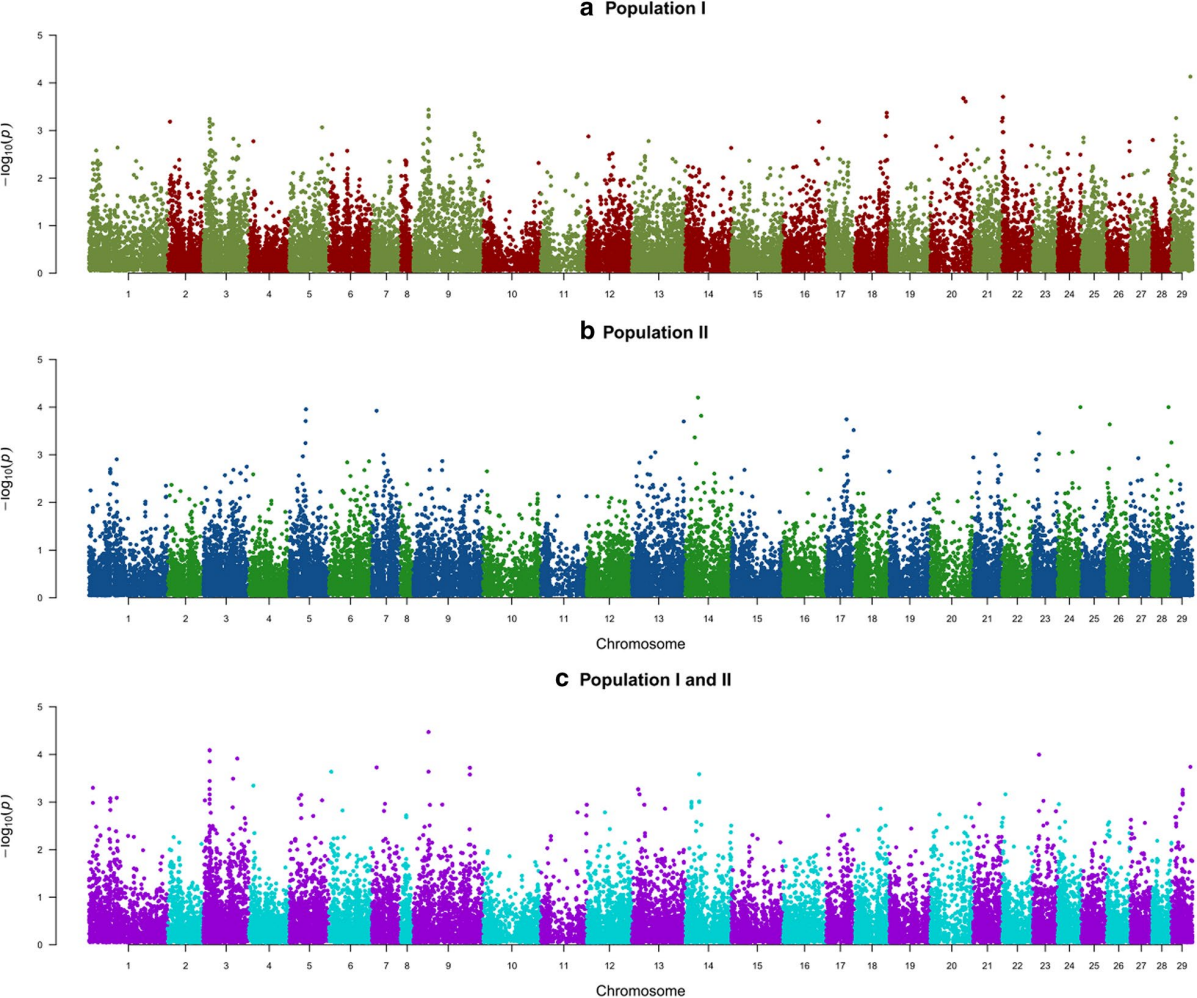
Manhattan plots of the genome-wide association study for populations I (**a**), II (**b**), and I and II combined (**c**). *Top markers* are close to chromosome-wide significance (α < 0.05) but do not pass the threshold

The results of the GWAS suggest that lice resistance is a polygenic trait, with no SNPs surpassing the Bonferroni-corrected significance thresholds (Fig. 1). Indeed, when each population was analysed separately, there was little overlap between regions that had the lowest P values (Fig. 1a, b). When the two populations were combined (Fig. 1c), SNPs with the lowest P values were located on chromosomes 1, 3, 9 and 23. The estimated proportion of additive genetic variance explained by these SNPs ranged from ~2 to 6 % each. The quantile–quantile (Q–Q) plots for each GWA analysis are in Figure S2 (See Additional file 2: Figure S2).

### Accuracy of predicted breeding values

**Fig. 2:**
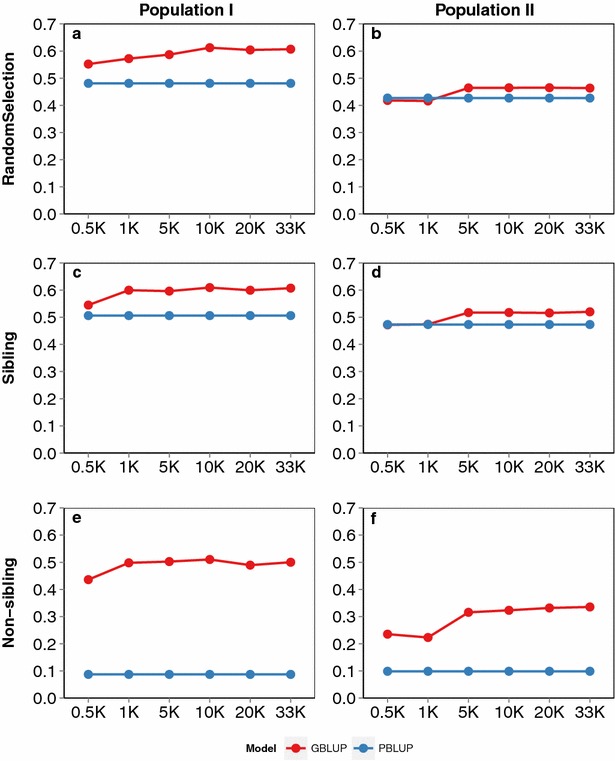
Accuracy of genomic and pedigree-based prediction within populations. Comparison of prediction accuracy (Y-axis) of two populations using increasing SNP densities from 0.5 to 33 K (X-axis) assessed by cross-validation analyses. “Random Selection” involved random assignment of individuals to training and validation sets (**a**) and (**b**); “Sibling” involved assigning full siblings from each family to both the training and validation sets (**c**) and (**d**); and “Non-sibling” involved avoidance of full-sibling animals in the training and validation sets (**e**) and (**f**). Panels **a**, **c** and **e** represent results for population I and panels **b**, **d**, and **f** represent those for population II

The putative polygenic architecture of lice resistance in these populations means that genomic prediction may be a practical and effective method of predicting breeding values for lice resistance, which was tested using cross-validation analyses under different scenarios in which varying SNP densities and varying levels of relatedness between training and validation sets were applied (see “Methods” for details). Accuracy of prediction using the genomic relationship matrix (GBLUP) was generally higher than that using the pedigree relationship matrix (PBLUP). Greater SNP density tended to improve prediction accuracy, but the asymptote was generally reached at ~5 K SNPs for both populations (Fig. 2).

The results of genomic prediction under the “random selection” (where training and validation sets were chosen at random), and “sibling” (where full siblings from each family were deliberately included in both the training and validation sets) scenarios were very similar for both populations (Fig. 2a–d). Therefore, including animals that share close relationships did not improve the accuracy of genomic predictions for these populations, which indicates that “random selection” will result in the presence of several closely-related fish across the training and validation datasets by chance. In both cases, GBLUP resulted in more accurate predictions of lice count in the validation data than PBLUP, with a relative advantage of approximately 27 % for population I and 10 % for population II (Fig. 2a–d). Increasing marker density to more than ~5 K randomly chosen SNPs had little impact on prediction accuracy, which may be expected when the training and validation sets are closely related [9].

**Fig. 3:**
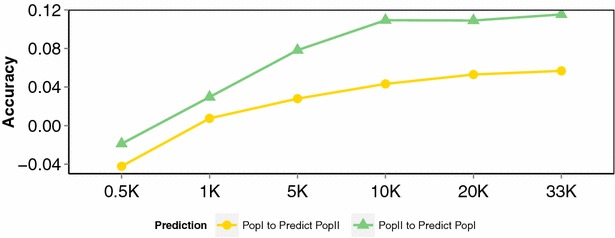
Accuracy of genomic prediction across populations. Based on setting population I as the training set and population II as the validation set and vice versa. Accuracy of prediction (Y-axis) for the two populations was estimated using increasing SNP density from 0.5 to 33 K (X-axis)

When the training and validation sets were less related, predictions of both pedigree- and genomic-based methods were less accurate, as expected. In the “non-sibling” scenario (where no full-siblings were included in both the training and validation sets), accuracies of prediction obtained with both GBLUP and PBLUP were substantially lower than those in the previous two scenarios. However, the benefit of genomic prediction was greatest under this scenario, with prediction accuracies increasing fivefold (population I) and 2.5-fold (population II) relative to pedigree-based prediction accuracies. Perhaps surprisingly, there was little benefit from increasing SNP density above ~5 K SNPs under this scenario as well (Fig. 2e, f). When the accuracy of genomic prediction was assessed across the two populations (corresponding to 2 year groups of the Landcatch broodstock), accuracies were markedly lower (0.05–0.11) than with all of the within-population scenarios (0.34–0.61). Increasing SNP density did seem to yield incremental (albeit small) increases in prediction accuracies when predicting across populations (Fig. 3), which suggested that this scenario was likely to benefit most from a high-density SNP array. However, these two populations were probably too small to achieve high prediction accuracy for these distantly-related animals, and a more thorough test of across-population prediction in salmon should use larger sample sizes.

## Discussion

Genomic selection is an increasingly important component of modern aquaculture breeding schemes, with simulated and applied studies highlighting its benefits over pedigree-based selection [9, 28]. However, the substantial cost of genome-wide genotyping means that the traits targeted by GS are likely to be those of high economic value, particularly those that cannot be easily measured on the selection candidates themselves. Currently, sea lice present the largest threat to the sustainability of salmon farming, which relies heavily on expensive and potentially environmentally-damaging chemical treatments [10]. Host resistance to sea lice has consistently been shown to have a substantial genetic component [11]. Therefore, resistance to lice is an ideal candidate trait for the application of GS. In our study, lice count data and genome-wide SNP genotypes were collected for two pedigreed salmon populations from a commercial breeding programme to assess the utility of genomic prediction of host resistance to sea lice under different scenarios, including a comparison to predictions based on pedigree records alone.

The heritability of resistance to lice was estimated at ~0.3 and 0.2 in populations I and II, respectively, which is similar to the findings of Gharbi et al. [11] (~0.3) and Gjerde et al. [29] (~0.2 to 0.3), and slightly higher than those of Ødegård et al. [9] (~0.13 to 0.14). However, it should be noted that the challenge experiments that are reported in Gharbi et al. [11], Gjerde et al. [13], and in our study, were all conducted in controlled tanks conditions, whereas the study of Ødegård et al. [9] was based on challenges in a sea-cage environment, which may display greater environmental variation. Furthermore, it should be noted that the higher heritability estimates for all traits in population I may be due in part to confounding between genetic and common environmental effects due to the family-specific rearing of the fry (compared to population II, for which individuals were mixed into a single tank as first feeding fry).

The GWAS indicated that host resistance to lice likely has a polygenic architecture, with no major QTL segregating in these populations (Fig. 1). Therefore, it is likely that individual QTL for lice resistance explain only a small percentage of the genetic variance, and a proportion of the QTL may be population-specific. As such, GBLUP and similar methods of genomic prediction are likely to be suitable for predicting breeding values for host resistance to lice, particularly within populations.

The degree of the genetic relationships between training and validation sets is critical for the efficacy of genomic prediction. In our study, genomic prediction was found to be highly effective and showed a significant advantage in terms of accuracy over pedigree-based methods within populations (which correspond to year groups of a salmon breeding programme, Fig. 2). The accuracy of prediction and the relative advantage of genomic prediction were lower for population II than for population I (Fig. 2), which may reflect the lower estimated heritability in this population because a low heritability can contribute to low prediction accuracy [20, 30]. Also, the family structure of population II was potentially less amenable to accurate prediction since it comprised a larger number of smaller families, which decreased the chance of having useful numbers of full siblings in the training and validation sets. Prediction accuracies were highest when training and validation sets were closely related, as was shown with the “Random selection” and “Sibling” scenarios. In addition, these results showed that deliberately including highly-related animals (i.e. full siblings) in the training and validation sets yielded little advantage over random assignment. This likely reflects the typical family structure of commercial salmon breeding populations, which consist of large full sibling families (thousands of fish per family) that result in close relationships between selection candidates and test individuals. However, the benefit of using genomic prediction over pedigree-based prediction was largest under the “Non-sibling” scenario, in which training and validation sets were established such that no full-siblings were included (i.e. the sets were less related than would be expected by chance, Fig. 2). Prediction across populations or year groups (for which genetic relationships are more distant) was substantially less effective, with relatively low prediction accuracies (Fig. 3). This may reflect, in part, inadequate sample size of the populations, or possibly differences in the experimental procedures between the two studies. However, our findings imply that either the GBLUP analyses did not efficiently capture short range LD between SNPs and QTL for resistance to sea lice, and/or that the QTL were population-specific. Therefore, in commercial salmon breeding schemes, regular phenotype data collection on animals that are closely-related to the selection candidates, combined with medium- or low-density (and cost) SNP panel genotyping appears to be the most effective means of using genomic prediction for resistance to lice. This strategy is supported by results from previous simulation studies (e.g. [28]).

Using data collected from a challenge trial performed in a sea cage environment, Ødegård et al. [9] also showed that genomic prediction of breeding values for lice resistance was more accurate compared to pedigree-based prediction. As in our study, the observed improvements depended partly on SNP density with ~32 (1 K SNPs) and 51 % (220 K SNPs) higher reliabilities than those obtained from predictions based on pedigree records alone [9]. Interestingly, increasing SNP density above a threshold of around 5 K SNPs had little impact on accuracy of prediction in both studies (Fig. 2, [9]). This may reflect the relatively close relationships between the training and validation sets, since higher SNP density did slightly improve the accuracy of cross-population predictions, as shown in our study, up to ~33 K SNPs (the highest density tested) (Fig. 3). However, it seems unlikely that linkage alone is underpinning the predictions, since predictions with low SNP densities (<1 K) and predictions based on an IBD (identity-by-descent) genomic relationship matrix were less accurate [9]. Therefore, short or long range LD between SNP and QTL alleles may be an important component of prediction. Obviously, such LD can be captured by a relatively sparse SNP set, a finding that may be related to the relatively close relationships between training and validation sets, recent population admixture [9], or slower decay of LD due to the lack of male recombination in male salmon across much of the genome [31, 32].

A difference between simulation studies and those performed on experimental data is often observed in genomic prediction studies. Previous simulation studies indicated that the accuracy of breeding value prediction can reach values of 0.8 to 1.0 if the reference population size is sufficiently large (e.g. more than 100,000) [2, 33]. However, in practice, due to financial and practical limitations, research programs that use ‘experimental’ data usually involve the analysis of relatively small reference populations [9, 21, 34]. It is likely that if we had used larger population sizes, higher accuracies of prediction would have been obtained, particularly for predictions across the two distantly-related populations (subject to sufficient SNP density). As such, cost-effective means of generating high-density SNP data remain a relevant goal, and genotype imputation is likely to be increasingly important, particularly now that the majority of the Atlantic salmon reference genome has been assembled and ordered onto chromosomes (Genbank assembly accession GCA_000233375.4, [35]). Genotyping-by-sequencing may be crucial for reaching such high SNP density at moderate cost and its potential for genomic prediction in livestock has already been reported [36]. With a high SNP density across large sample sizes, one may expect to capture LD between SNPs and QTL, and co-segregation of chromosome segments among related individuals, although the resolution of mapping causative variants may be hampered by the strong relationship structure in the population. Within populations/year groups, the requirement in terms of SNP density for accurate prediction is clearly lower and as few as 1 to 5 K informative SNPs are sufficient. However, while this points to the potential utility of cheaper and lower density genotyping platforms in aquaculture breeding, it is important to keep in mind that SNP informativeness can vary between populations.

## Conclusions

Genomic prediction is an effective method for predicting breeding values for host resistance to sea lice in Atlantic salmon populations from a commercial breeding programme. The GWAS results suggested that lice resistance is a polygenic trait. Cross-validation tests of genomic prediction highlighted the substantial improvements in prediction accuracy compared to that of pedigree-based prediction. The accuracy of GBLUP was highest when training and validation sets were closely related but the relative advantage over pedigree-based prediction within a population was largest when relationships were more distant. Relatively low SNP densities (from 1 to 5 K SNPs) were sufficient for accuracy to reach the asymptote under most of the scenarios tested. Prediction accuracy is generally much lower across distantly-related populations, although a trend was evident that increased marker density was advantageous in such situations. Therefore, larger population sample sizes and high-density SNP genotypes are probably necessary to improve across-population prediction. Given the economic importance of resistance to sea lice, and the efficacy of genomic prediction, it is likely that selective breeding for this trait using genomic data will become an important component of sea lice control.

## Additional files

10.1186/s12711-016-0226-9 Distributions of data for lice counts and after data normalization. Panels (a) and (c) represent results for population I, and panel (b) and (d) represent results for population II.
10.1186/s12711-016-0226-9 Quantile-quantile (Q-Q) plot for the GWAS analysis. Description: Three Q-Q plots are given in the file including population I (A), population II (B) and populations I and II combined (C).

## References

[1] Habier D, Fernando RL, Garrick DJ (2013). Genomic BLUP decoded: a look into the black box of genomic prediction. Genetics.

[2] Meuwissen THE, Hayes BJ, Goddard ME (2001). Prediction of total genetic value using genome-wide dense marker maps. Genetics.

[3] Wilkinson S, Wiener P, Archibald AL, Law A, Schnabel RD, McKay SD (2011). Evaluation of approaches for identifying population informative markers from high density SNP chips. BMC Genet.

[4] Riggio V, Matika O, Pong-Wong R, Stear MJ, Bishop SC (2013). Genome-wide association and regional heritability mapping to identify loci underlying variation in nematode resistance and body weight in Scottish Blackface lambs. Heredity (Edinb).

[5] Bermingham ML, Bishop SC, Woolliams JA, Pong-Wong R, Allen AR, McBride SH (2014). Genome-wide association study identifies novel loci associated with resistance to bovine tuberculosis. Heredity (Edinb).

[6] Gutierrez AP, Yáñez JM, Fukui S, Swift B, Davidson WS (2015). Genome-wide association study (GWAS) for growth rate and age at sexual maturation in Atlantic salmon (*Salmo salar*). PLoS One.

[7] Yáñez JM, Newman S, Houston RD (2015). Genomics in aquaculture to better understand species biology and accelerate genetic progress. Front Genet.

[8] Daetwyler HD, Pong-Wong R, Villanueva B, Woolliams JA (2010). The impact of genetic architecture on genome-wide evaluation methods. Genetics.

[9] Odegård J, Moen T, Santi N, Korsvoll SA, Kjøglum S, Meuwissen THE (2014). Genomic prediction in an admixed population of Atlantic salmon (*Salmo salar*). Front Genet.

[10] Frazer LN, Morton A, Krkosek M (2012). Critical thresholds in sea lice epidemics: evidence, sensitivity and subcritical estimation. Proc Biol Sci.

[11] Gharbi K, Matthews L, Bron J, Roberts R, Tinch A, Stear MJ (2015). The control of sea lice in Atlantic salmon by selective breeding. J R Soc Interface.

[12] Kolstad K, Heuch PA, Gjerde B, Gjedrem T, Salte R (2005). Genetic variation in resistance of Atlantic salmon (*Salmo salar*) to the salmon louse *Lepeophtheirus salmonis*. Aquaculture.

[13] Gjerde B, Ødegård J, Thorland I (2011). Estimates of genetic variation in the susceptibility of Atlantic salmon (*Salmo salar*) to the salmon louse *Lepeophtheirus salmonis*. Aquaculture.

[14] Houston RD, Bishop SC, Guy DR, Tinch AE, Taggart JB, Bron JE, et al. Genome wide association analysis for resistance to sea lice in Atlantic salmon: application of a dense SNP array. In: Proceedings of the 10th World congress of genetics applied to livestock production: 17–22 August 2014; Vancouver. 2014. https://asas.org/docs/default-source/wcgalp-proceedings-oral/265_paper_9597_manuscript_751_0.pdf?sfvrsn=2.

[15] Habier D, Fernando RL, Kizilkaya K, Garrick DJ (2011). Extension of the bayesian alphabet for genomic selection. BMC Bioinformatics.

[16] Daetwyler HD, Calus MPL, Pong-Wong R, de los Campos G, Hickey JM (2013). Genomic prediction in animals and plants: Simulation of data, validation, reporting, and benchmarking. Genetics.

[17] Habier D, Fernando RL, Dekkers JCM (2007). The impact of genetic relationship information on genome-assisted breeding values. Genetics.

[18] Wientjes YCJ, Veerkamp RF, Calus MPL (2013). The effect of linkage disequilibrium and family relationships on the reliability of genomic prediction. Genetics.

[19] Hickey JM, Dreisigacker S, Crossa J, Hearne S, Babu R, Prasanna BM (2014). Evaluation of genomic selection training population designs and genotyping strategies in plant breeding programs using simulation. Crop Sci.

[20] Vela-Avitúa S, Meuwissen THE, Luan T, Ødegård J (2015). Accuracy of genomic selection for a sib-evaluated trait using identity-by-state and identity-by-descent relationships. Genet Sel Evol.

[21] Tsai HY, Hamilton A, Tinch AE, Guy DR, Gharbi K, Stear MJ (2015). Genome wide association and genomic prediction for growth traits in juvenile farmed Atlantic salmon using a high density SNP array. BMC Genomics.

[22] Houston RD, Taggart JB, Cézard T, Bekaert M, Lowe NR, Downing A (2014). Development and validation of a high density SNP genotyping array for Atlantic salmon (*Salmo salar*). BMC Genomics.

[23] Purcell S, Neale B, Todd-Brown K, Thomas L, Ferreira MAR, Bender D (2007). PLINK: a tool set for whole-genome association and population-based linkage analyses. Am J Hum Genet.

[24] Gilmour AR, Gogel BJ, Cullis BR, Thompson R. ASReml user guide. 4th ed. Hemel Hempstead: VSN International Ltd; 2014.

[25] Aulchenko YS, Ripke S, Isaacs A, van Duijn CM (2007). GenABEL: an R library for genome-wide association analysis. Bioinformatics.

[26] VanRaden PM (2008). Efficient methods to compute genomic predictions. J Dairy Sci.

[27] Chen WM, Abecasis GR (2007). Family-based association tests for genomewide association scans. Am J Hum Genet.

[28] Sonesson AK, Meuwissen THE (2009). Testing strategies for genomic selection in aquaculture breeding programs. Genet Sel Evol.

[29] Gjerde B, Pante MJR, Baeverfjord G (2005). Genetic variation for a vertebral deformity in Atlantic salmon (S*almo salar*). Aquaculture.

[30] Nirea KG, Sonesson AK, Woolliams JA, Meuwissen THE (2012). Strategies for implementing genomic selection in family-based aquaculture breeding schemes: double haploid sib test populations. Genet Sel Evol.

[31] Lien S, Gidskehaug L, Moen T, Hayes BJ, Berg PR, Davidson WS (2011). A dense SNP-based linkage map for Atlantic salmon (*Salmo salar*) reveals extended chromosome homeologies and striking differences in sex-specific recombination patterns. BMC Genomics.

[32] Gonen S, Lowe NR, Cezard T, Gharbi K, Bishop SC, Houston RD (2014). Linkage maps of the Atlantic salmon (*Salmo salar*) genome derived from RAD sequencing. BMC Genomics.

[33] Daetwyler HD, Villanueva B, Woolliams JA (2008). Accuracy of predicting the genetic risk of disease using a genome-wide approach. PLoS One.

[34] Calus MPL (2010). Genomic breeding value prediction: methods and procedures. Animal.

[35] Davidson WS, Koop BF, Jones SJM, Iturra P, Vidal R, Maass A (2010). Sequencing the genome of the Atlantic salmon (*Salmo salar*). Genome Biol.

[36] Gorjanc G, Cleveland MA, Houston RD, Hickey JM (2015). Potential of genotyping-by-sequencing for genomic selection in livestock populations. Genet Sel Evol.

