# Supplementary figures and images for: Genomic prediction of host resistance to sea lice in farmed Atlantic salmon populations
Source: Genet Sel Evol. 2016 Jun 29;48:47. doi: 10.1186/s12711-016-0226-9 (PMC4926294; doi:10.1186/s12711-016-0226-9)

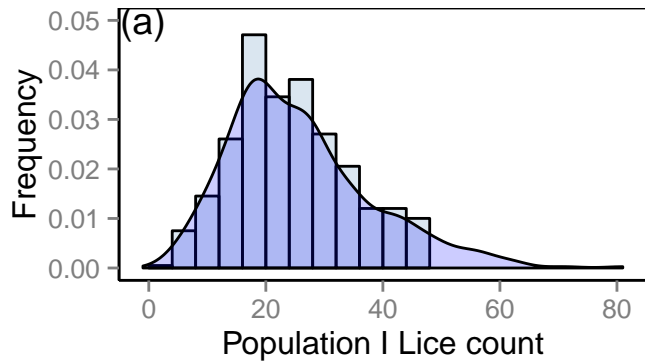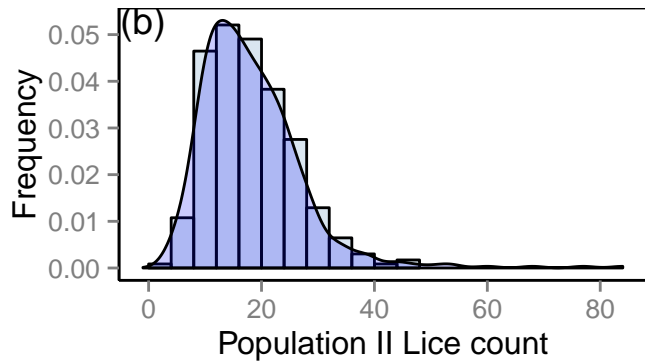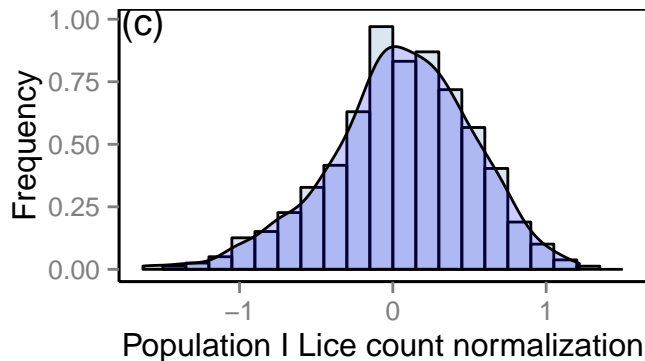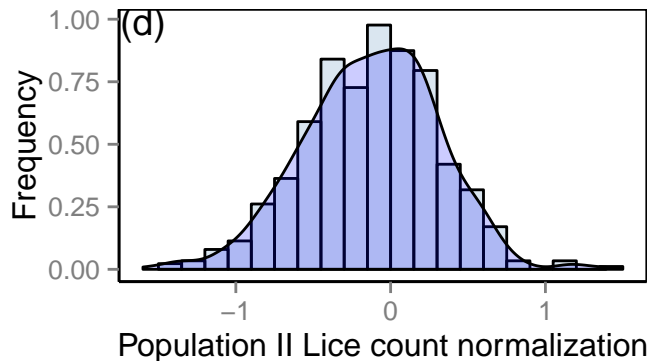

Supplement: Supplementary file 1 — 10.1186/s12711-016-0226-9 Distributions of data for lice counts and after data normalization. Panels (a) and (c) represent results for population I, and panel (b) and (d) represent results for population II. [file 12711_2016_226_MOESM1_ESM.pdf]
