# Supplementary material for: Genomic prediction of host resistance to sea lice in farmed Atlantic salmon populations
Source: Genet Sel Evol. 2016 Jun 29;48:47. doi: 10.1186/s12711-016-0226-9 (PMC4926294; doi:10.1186/s12711-016-0226-9)

Additional file 2. The quantile-quantile (Q-Q) plot for the GWA analyses.

(A) Population I

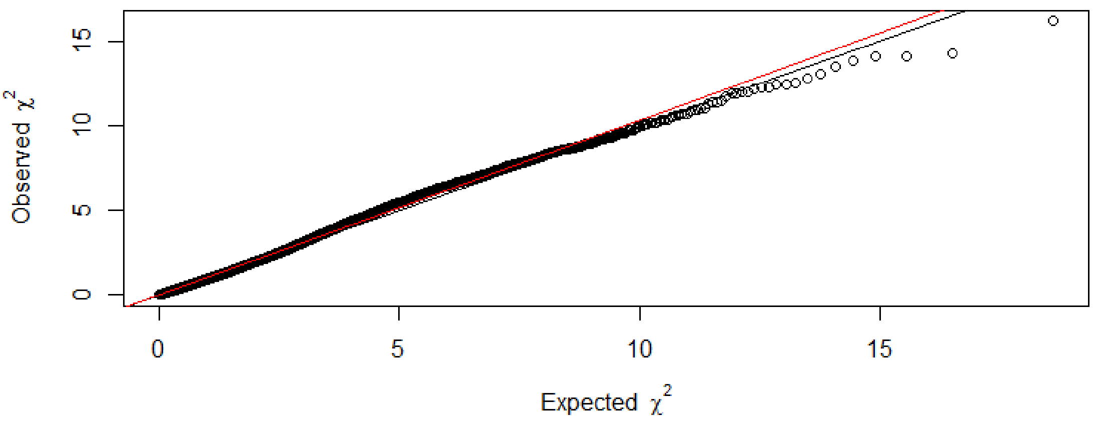

(B) Population II

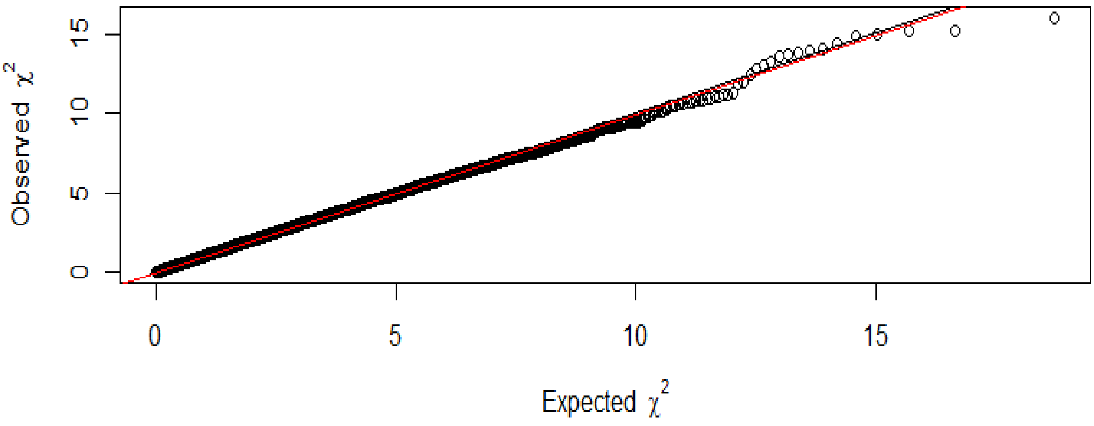

(C) Populations I and II combined

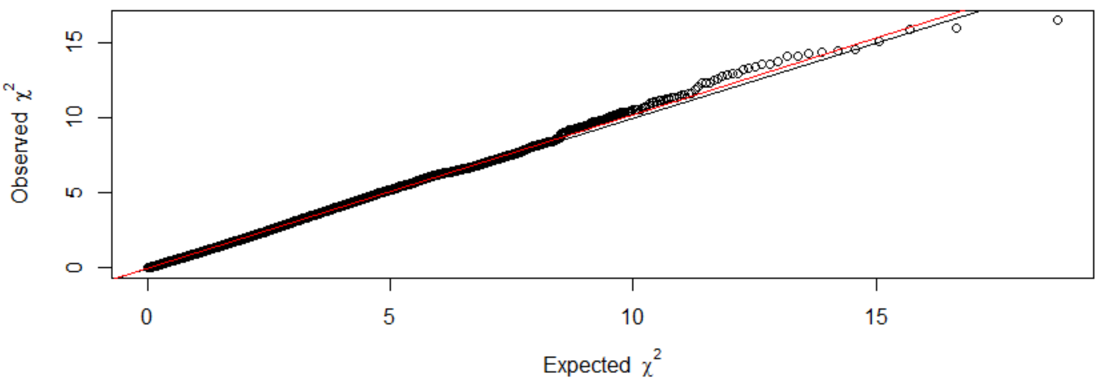

Supplement: Supplementary file 2 — 10.1186/s12711-016-0226-9 Quantile-quantile (Q-Q) plot for the GWAS analysis. Description: Three Q-Q plots are given in the file including population I (A), population II (B) and populations I and II combined (C). [file 12711_2016_226_MOESM2_ESM.pdf]
